# Sphingosine-1-phosphate Signalling in Aneurysmal Subarachnoid Haemorrhage: Basic Science to Clinical Translation

**DOI:** 10.1007/s12975-023-01133-9

**Published:** 2023-02-07

**Authors:** Ben Gaastra, John Zhang, Will Tapper, Diederik Bulters, Ian Galea

**Affiliations:** 1 Faculty of Medicine, University of Southampton, Southampton, SO17 1BJ UK; 2 Department of Neurosurgery, Wessex Neurological Centre, University Hospital Southampton, Southampton, SO16 6YD UK; 3 Center of Neuroscience Research, Loma Linda University, Loma Linda, CA 92350 USA

**Keywords:** Subarachnoid haemorrhage, Sphingosine-1-phosphate, Outcome, Sphingosine-1-phosphate receptor modulators

## Abstract

Sphingosine-1-phosphate (S1P) is generated intracellularly and, when transported to the extracellular compartment, predominantly signals through S1P receptors. The S1P signalling pathway has been implicated in the pathophysiology of neurological injury following aneurysmal subarachnoid haemorrhage (aSAH). In this review, we bring together all the available data regarding the role of S1P in neurological injury following aSAH. There is agreement in the literature that S1P increases in the cerebrospinal fluid following aSAH and leads to cerebral artery vasospasm. On the other hand, the role of S1P in the parenchyma is less clear cut, with different studies arguing for beneficial and deleterious effects. A parsimonious interpretation of this apparently conflicting data is presented. We discuss the potential of S1P receptor modulators, in clinical use for multiple sclerosis, to be repurposed for aSAH. Finally, we highlight the gaps in our knowledge of S1P signalling in humans, the clinical challenges of targeting the S1P pathway after aSAH and other research priorities.

## Introduction

Aneurysmal subarachnoid haemorrhage (aSAH) is a rare but devastating form of stroke caused by the rupture of a cerebral artery aneurysm into the subarachnoid space. aSAH is associated with significant morbidity and mortality [1]. It affects younger individuals compared to other stroke types and consequently has the highest socio-economic impact of any form of stroke [2].

Neurological injury following aSAH can be considered in two phases. Early brain injury (EBI), occurring within 72 h, is a consequence of the initial surge in intracranial pressure and subsequent reduction in cerebral blood flow caused by the haemorrhage. Cascades initiated by EBI and the presence of blood and its breakdown products within the subarachnoid space lead to a delayed brain injury characterised by inflammation, oxidative injury, microthrombosis, cerebral vasospasm and abnormal cortical electrical activity [3–7]. These pathological processes are driven by a wide range of signalling pathways, and there is a growing body of evidence that sphingolipids and sphingosine-1-phosphate (S1P) act as key signalling molecules in these pathways.

**Fig. 1:**
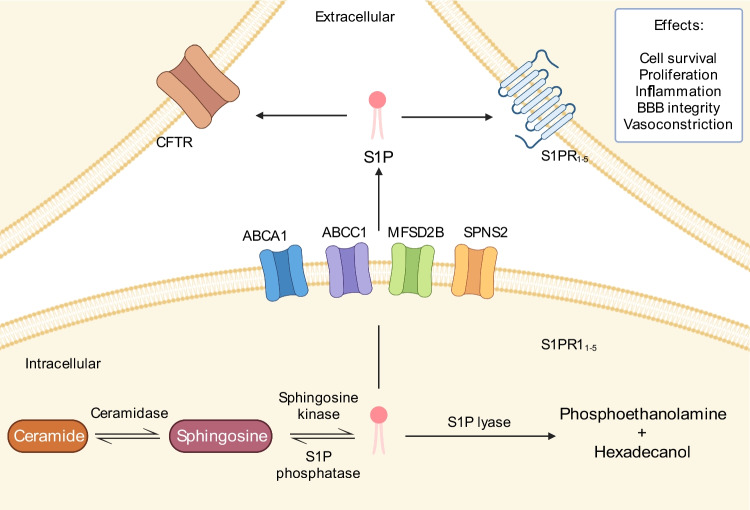
Sphingosine-1-phosphate (S1P) signalling pathway. Sphingosine is generated when ceramide is degraded by ceramidase. S1P is synthesised intracellularly via reversible phosphorylation of sphingosine by sphingosine kinase. Intracellularly, S1P can be dephosphorylated back to sphingosine by the action of S1P phosphatase or is degraded to phosphoethanolamine and hexadecanol by the action of S1P lyase. S1P is transported to the extracellular compartment by a number of transporters including ATP-binding cassette (ABC) transporters A1 and C1, MFSD2B and sphingolipid transporter 2 (SPNS2). In the extracellular compartment, S1P can be transported back into cells via the cystic fibrosis transmembrane conductance receptor (CFTR) or signal through S1P receptors (S1PR_1_–S1PR_5_). In the context of subarachnoid haemorrhage, S1PR signalling influences cell survival, proliferation, inflammation and blood-brain barrier (BBB) integrity and causes cerebral artery vasoconstriction. Created with BioRender.com

Sphingosine is generated when ceramide is degraded by ceramidase. S1P is synthesised intracellularly via reversible phosphorylation of sphingosine by sphingosine kinases 1 and 2 (SphK1 and SphK2, respectively). Intracellularly, S1P can be dephosphorylated back to sphingosine by the action of phosphatases (SPP1 and SPP2) or is degraded to phosphoethanolamine and hexadecanol by the action of S1P lyase (SPL). S1P levels, therefore, depend on the balance of these metabolic enzymes [8, 9]. S1P is transported to the extracellular compartment by a number of transporters including ATP-binding cassette (ABC) transporters A1 [10] and C1 [11], MFSD2B [12] and sphingolipid transporter 2 (SPNS2) [13] (Fig. 1). S1P is predominantly found in the plasma and lymph with the primary source being red blood cells supplemented by vascular endothelium, lymphatic endothelium and activated platelets [8]. Additionally, in the central nervous system (CNS), the arachnoid membrane acts as a source of S1P [14].

In the extracellular compartment, S1P can be transported back into cells via the cystic fibrosis transmembrane conductance receptor (CFTR) [15] or signal through S1P receptors (S1PRs). S1P can inhibit CFTR activity which may reinforce S1P signalling via S1PRs [16], although it remains to be seen whether this occurs at S1P concentrations encountered in the cerebrospinal fluid (CSF) after aSAH. There are five S1PR subtypes (S1PR_1_–S1PR_5_) [17] expressed mainly in the cardiovascular, immune [18] and central nervous systems (Table 1). S1P plays a role in vascular development and function including maintenance of vascular integrity and regulation of tone through action on vascular smooth muscle cells. Elevated levels of S1P in the plasma and lymph play a key role in the immune system by promoting trafficking of lymphocytes from lymphoid tissue to the circulation up a S1P concentration gradient [8]. Within the brain, S1P signalling has been implicated in a number of key processes including cell survival [19], proliferation [20], inflammation [17], vasoconstriction [21] and blood-brain barrier (BBB) integrity [22, 23]. With this broad range of functions, S1P signalling has been linked to a number of neurological conditions including stroke and aSAH [9].

S1PRs have been reviewed in detail elsewhere [18, 24]. The five receptor subtypes are G protein-coupled receptors and are differentially expressed within the CNS according to cell type [18]. It is useful to think of S1PR in terms of expression by cells in two locations: within the brain parenchyma and the vascular tree (Table 1). S1PR_1_ is expressed primarily on neural progenitor cells, astrocytes and oligodendrocytes. S1PR_2_ and S1PR_3_ are expressed on neurons, astrocytes and microglia, with S1PR_3_ also expressed on oligodendrocytes. S1PR_4_ is not expressed significantly in the CNS. S1PR_5_ is primarily expressed on oligodendrocytes. In addition, S1PR_1_–S1PR_3_ are expressed in the cardiovascular system (on atrial myocytes, endothelial cells and smooth muscle cells [18]) including in the CNS vasculature. All subtypes (S1PR_1_–S1PR_5_) are expressed in cells of the immune system (on lymphocytes, natural killer cells, monocytes and dendritic cells [18]) [8, 38]. S1P may also act as a ligand for other receptors including triggering receptor expressed on myeloid cell 2 (TREM2) which will be discussed further below [39].

**Table 1:** Summary of S1PR subtype expression in the CNS, role at baseline and after SAH, targeting modulators and experimental antagonists referenced in this review. References specifically relate to evidence in SAH

Receptor subtype	CNS expression	Role		S1PR modulator	Experimental antagonists used in SAH
		Baseline	SAH specific		
S1PR_1_	Neural progenitor cells Astrocytes Oligodendrocytes CNS-infiltrating leucocytes Blood vessels	Cell survival and function Endothelial cell function	Maintain BBB integrity, reduce brain oedema, anti-inflammatory (< 24 h) [25–29] Pro-inflammatory astrocytic activation (48–72 h) [30]	Fingolimod [31, 32] Siponimod Ozanimod Ponesimod [30]	VPC23019 [25–29, 33] JTE013 [21] RP001 hydrochloride [34]
S1PR_2_	Neurons Astrocytes Microglia CNS-infiltrating leucocytes Blood vessels	Endothelial cell function Vascular tone	–	–	JTE013 [21]
S1PR_3_	Neurons Astrocytes Microglia Oligodendrocytes CNS-infiltrating leucocytes Blood vessels	Cell survival and function Endothelial cell function	Maintain BBB integrity, reduce brain oedema, anti-inflammatory (< 24 h) [25–28] Cerebral artery vasoconstriction [14, 35, 36]	Fingolimod [31, 32]	TY 52156 [14] VPC23019 [25–29, 33] CAY10444 [37] JTE013 [21] RP001 hydrochloride [34]
S1PR_4_	CNS-infiltrating leucocytes	–	–	Fingolimod [31, 32]	JTE013 [21] RP001 hydrochloride [34]
S1PR_5_	Oligodendrocytes CNS-infiltrating leucocytes	Cell survival and myelination	–	Fingolimod [31, 32] Siponimod Ozanimod	JTE013 [21] RP001 hydrochloride [34]

*BBB* blood-brain barrier, *CNS* central nervous system, *S1PR* spingosine-1-phosphate receptor, *SAH* subarachnoid haemorrhage

## S1P Signalling after aSAH

**Fig. 2:**
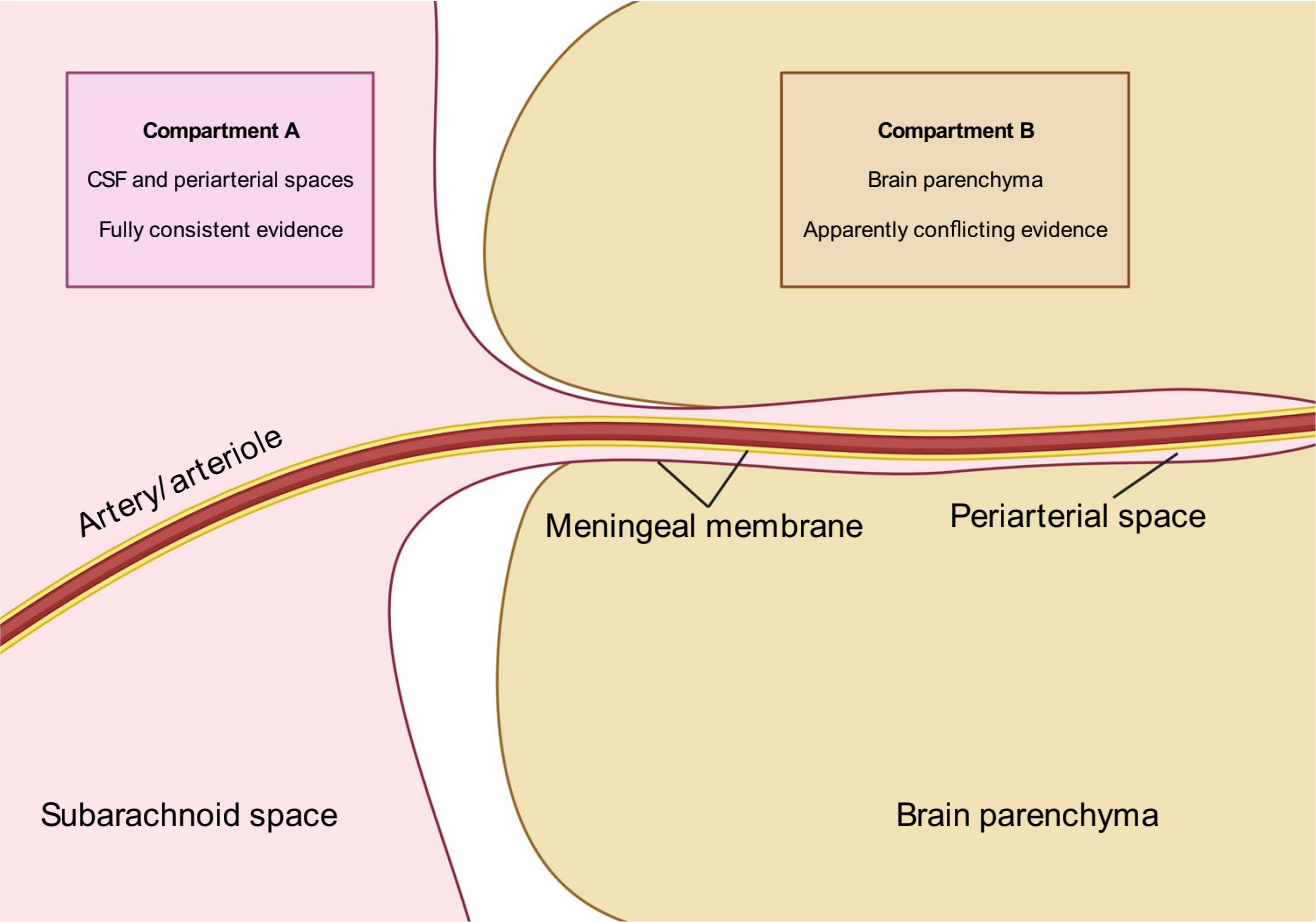
The evidence supporting the action of sphingosine-1-phosphate is fully consistent within the cerebrospinal fluid (CSF) and periarterial spaces (compartment A, pink colour). Though evidence appears to be conflicting within the parenchyma (compartment B, gold colour), there are likely to be reasons for this, as discussed in the text. Created with BioRender.com

S1P signalling has been extensively implicated in neurological injury following stroke, and there is a growing body of evidence from animals and humans that it also plays a key role in the pathophysiology of aSAH (Table 1). Specifically, a picture is starting to emerge suggesting that S1P may have opposing effects in two different CNS compartments: CSF and brain parenchyma (Fig. 2). Here, we introduce the spatial distinction between the two different compartments alongside its supporting evidence. This distinction is important since although there is agreement regarding the detrimental effect of S1P in the CSF, the literature is apparently conflicting within the brain parenchyma (Fig. 2), and we here try to reconcile these discrepancies.

### Cerebrospinal Fluid

Männer et al. [40] showed that S1P levels were elevated in the CSF of aSAH cases (*n* = 18) compared to controls. Levels were highest in day 1, then decreased over 2 weeks. They also demonstrated that S1P levels in the CSF correlated with haemorrhage volume. Taken together, this strongly suggests that the blood clot is the primary source/stimulus for CSF S1P, especially since red blood cells and platelets are cells which harbour and/or produce large amounts of S1P. The same study also implicated S1P levels in outcome by showing a trend towards higher CSF S1P in cases with symptomatic vasospasm and a significant association of CSF S1P with outcome (assessed by the modified Rankin scale) at 12 months using a sliding scale, but not when dichotomised [40].

CSF bathes cerebral arteries and accompanies arterioles penetrating the base of the brain along periarterial spaces [41] (Fig. 2). Consequently, CSF S1P may be able to influence both macrovascular and microvascular vasoconstriction and, ultimately, neurological outcome. S1P has been shown in vitro to induce vasoconstriction in mouse middle cerebral arteries [14] and both in vitro and in vivo in canine basilar arteries [42]. S1P expression has also been demonstrated to be elevated in vasospastic vessels from a rat model of SAH [43]. In keeping with this, vascular tone was reduced in SphK2 knock-out mouse cerebral vessels with decreased S1P signalling [14]. S1P-dependent vasoconstriction has been linked to outcome since non-specific S1PR antagonism (with JTE013) reduced cerebral artery vasoconstriction and neuronal apoptosis and improved neurological outcomes in a mouse model of SAH [21]. In a post-mortem analysis of a single human, who had not suffered aSAH, S1P was localised to the media and adventitial layer of the middle cerebral artery supporting a role in vascular reactivity specifically in humans [14]. Overall, this suggests that elevated levels of S1P in the CSF following aSAH may influence outcome by stimulating vasoconstriction.

S1PR_3_ appears to play an important role in S1P-mediated cerebral artery vasoconstriction. Using rat cerebral artery and aortic vascular smooth muscle cells, Coussin et al. [35] showed that S1P induced much greater vasoconstriction in cerebral arteries compared to the aorta. S1PR_1_ was equally expressed in the cerebral artery and aortic vascular smooth muscle cells, but S1PR_2_ and S1PR_3_ showed higher expression in the cerebral artery vascular smooth muscle cells, suggesting an important role for these receptors in S1P-mediated vasoconstriction [35]. A subsequent study showed S1P-induced vasoconstriction in wild-type and S1PR_2_ null but not in S1PR_3_ null mice, emphasising the importance of S1PR_3_ [36]. The same study also demonstrated that a selective S1PR_1_ agonist (SEW2871) did not cause vasoconstriction in rat basilar arteries [36]. Finally, a specific S1PR_3_ antagonist (TY 52156) significantly reduced S1P-induced mouse middle cerebral artery vasoconstriction in vitro [14]. Together, these studies support a key role for S1PR_3_ in vasoconstriction. However, it is still possible that other S1PRs play a role since antagonists are not always as specific as thought and knock-out mice may develop compensatory changes during development. Also, data is emerging that brain microvascular endothelial cells express S1PR_4_ and S1PR_5_ [44].

Further evidence linking S1P to cerebral artery vasoconstriction comes from studies of CFTR expression on vascular smooth muscle cells. S1P can be dephosphorylated by SPP, and therefore, S1P levels are influenced by the balance of SphK and SPP activity. SPP is intracellular and relies on S1P being transported into cells prior to action, a process dependent on CFTR [15] (Fig. 1). After SAH, cerebral artery CFTR protein expression was found to be downregulated via tumour necrosis factor alpha, and pharmacological inhibition of this cytokine improved neuronal survival and functional outcome [21]. Since CFTR downregulation would be expected to contribute to higher extracellular S1P, this suggested that CFTR was mediating outcome via S1P. Further evidence supporting this hypothesis came from experiments with mice homozygous for the ΔF508 CFTR mutation (expected to increase extracellular S1P levels) which showed increased vascular tone and decreased cerebral blood flow [45]. In vitro, CFTR downregulation in vascular smooth muscle cells reduced cellular uptake of S1P (i.e. relatively increased extracellular S1P levels), and S1P uptake was normalised by the CFTR corrector compound C18. In the same study using ΔF508 CFTR mice, upregulation of CFTR function using the CFTR corrector compound C18 or lumacaftor improved cerebral blood flow and reduced neuronal injury in a mouse model of SAH [45]. CFTR is also expressed on neurons [46] although the role of neuronal CFTR in S1P metabolism has not been explored after aSAH.

Overall, this body of evidence suggests that S1P is elevated in the CSF following aSAH and may influence outcome by causing cerebral artery vasoconstriction, a process known to be associated with neurological injury. S1P-mediated vasoconstriction appears dependent on S1PRs (likely S1PR_3_ in particular), and therefore, this pathway may be a potential therapeutic target. A limitation of this evidence is that it is predominantly derived from small animal studies. Although phylogenetic analysis shows that S1PRs are conserved between vertebrate species, this does not necessarily mean that receptor functions are similar between rodents and humans. As an example, the chronotropic cardiac effects of S1PR modulators are mediated by S1PR_3_ in mice, but by S1PR_1_ in humans [38, 47]. Clearly, further investigation of S1P-mediated vasoconstriction in humans is required.

**Fig. 3:**
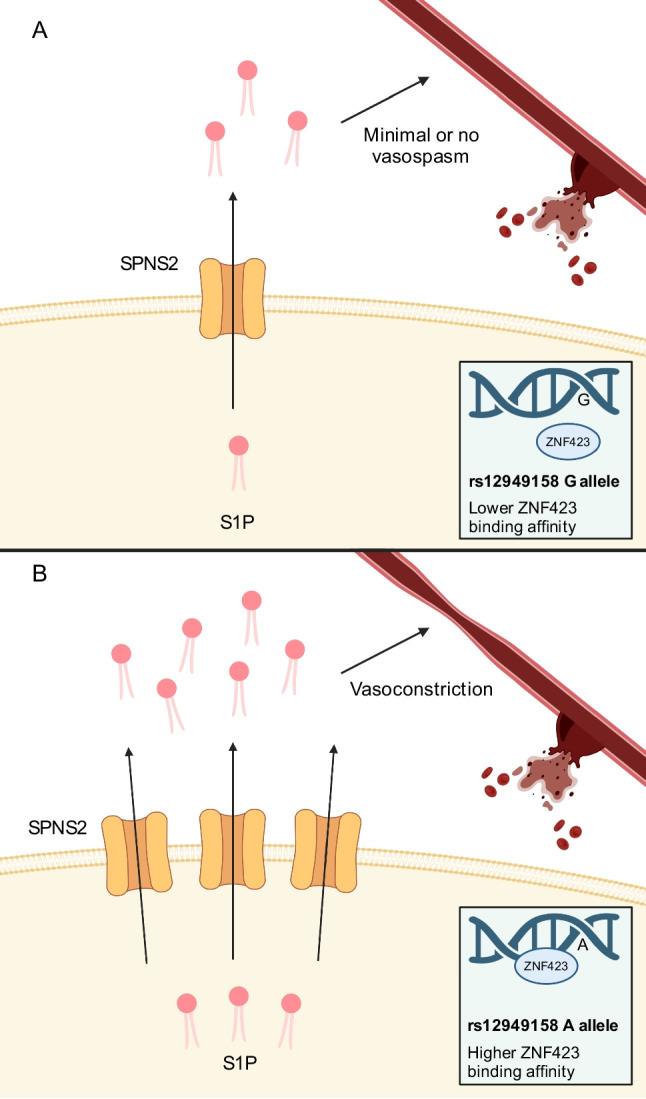
Proposed mechanism by which the rs12949158 single nucleotide polymorphism in the SPNS2 gene may be associated with poor outcome after aneurysmal subarachnoid haemorrhage. **A** SPNS2 is a sphingosine-1-phosphate (S1P) exporter. Within the SPNS2 gene, the intronic rs12949158 variant (reference allele G) is located within the binding site for the transcription factor ZNF423. **B** The alternate rs12949158 A allele is associated with significantly increased ZNF423 transcription factor binding affinity. Increased transcription factor binding leads to upregulation of SPNS2, increased S1P export from cells into the cerebrospinal fluid and thereby a higher S1P concentration in the subarachnoid space. S1P binds to S1PR_3_ expressed by cerebral artery smooth muscle, leading to worse neurological outcome. Created with BioRender.com

In keeping with the above evidence, a recent genome-wide meta-analysis (*n* = 2489) identified that the rs12949158 variant in the SPNS2 gene was associated with dichotomised clinical outcome after aSAH (*p* = 4.29 × 10^−8^) [48]. The risk of poor outcome was estimated to be 2.15-fold higher (95% confidence interval 1.63–2.82) in patients with the risk allele (A). rs12949158 is an intronic variant in SPNS2 located within the ZNF423 transcription factor binding site. The rs12949158 alternate A allele is associated with a significant increase in transcription factor binding affinity (*p* = 5.5 × 10^−13^), as predicted by HaploReg [49] using a positional weight matrix–based algorithm [50]. A potential mechanism by which the alternate rs12949158 allele could influence outcome after aSAH is through increased binding affinity of ZNF423, resulting in upregulation of SPNS2 and an increase in S1P released into the CSF, leading to cerebral artery vasoconstriction and neurological injury (Fig. 3). As SPNS2 is expressed in the CNS including on microglia [51] and endothelial cells, where it plays a key role in the blood-brain barrier [23], it is well placed to influence CSF S1P levels. This finding awaits confirmation since the analysis was limited by sample size and outcome metric heterogeneity. While validation of SPNS2 as a critical genetic marker of outcome is required in a larger cohort, the finding supports a potentially pivotal role for S1P signalling following aSAH in humans.

### Brain Parenchyma

#### A Beneficial Role for S1P/S1PR Signalling in the Parenchyma

Using rat brain homogenates, Testai et al. [52] showed a decrease in S1P and an increase in ceramide levels in the parenchyma at 48 h following experimental SAH. They suggested that this may represent a shift in sphingolipid metabolism away from S1P after SAH [52]. It is not clear whether this decrease in parenchymal S1P occurred in the intracellular or extracellular space or both. It is interesting to note that in the early stages after SAH, while S1P is raised in the CSF as a result of the blood clot, the concentration of S1P decreases in the parenchyma. However, we do not know what happens to S1P in the parenchyma after 48 h, when delayed vasospasm occurs. It is possible that parenchymal S1P levels rise, as a result of either CSF mixing with interstitial fluid or local synthesis, such that parenchymal S1P levels contribute to delayed microvascular spasm.

A number of studies focussing on early time points have suggested that upregulation of S1P in the parenchyma may have a neuroprotective effect. In a series of studies, Altay et al. [25–28] assessed the impact of the volatile anaesthetic agents isoflurane and sevoflurane in a mouse endovascular perforation model of SAH. Over four published studies, this group demonstrated that 2% isoflurane and 3% sevoflurane given 1 h after induction of SAH, over a period of another hour, decreased brain oedema, BBB disruption, neuro-inflammation (including COX-2 and cytokine expression) and neuronal death, and improved neurological outcome [25–28]. Using brain homogenates, they demonstrated elevated levels of parenchymal SphK2 and postulated that the effect of these anaesthetic agents was mediated by increased S1P levels. This hypothesis was supported by showing that the effect of these anaesthetic agents was reduced by inhibiting SphK (using *N*,*N*-dimethylsphingosine) and antagonising S1PR_1_ and S1PR_3_ (using VPC23019). Only one of these studies evaluated brain oedema and neurological outcome beyond 24 h, and it showed that neither was improved at 72 h after experimental SAH [26].

A further study by the same group using low-dose subcutaneous heparin 2 h after SAH induction in the same mouse model showed a neuroprotective effect at 24 h, assessed by neurological score, brain water content and neuronal death. This was associated with an increase in SphK1 levels in brain homogenate, leading to the indirect implication that parenchymal S1P is beneficial after SAH [53]. This was supported by a separate study using the anti-malarial artesunate in a rat endovascular perforation model of SAH [29]. In this study, administration of daily artesunate reduced neurological impairment and brain oedema up to 72 h. S1P signalling in the brain parenchyma was indirectly implicated as S1PR_1_ expression in brain homogenates was increased by artesunate at 24 h, but other S1PR subtypes were not studied. The clinical neurological benefits of artesunate were attenuated after S1PR_1_ downregulation (with short interfering RNA) or S1PR_1_ and S1PR_3_ antagonism (with VPC23019). In this study, however, SphK inhibition (using *N*,*N*-dimethylsphingosine) did not reverse the effects of artesunate, suggesting that upregulation of S1PR_1_ rather than S1P itself mediated the neurological effect of artesunate [29].

S1P has recently been shown to act via the TREM2 receptor to promote microglial phagocytosis of apoptotic neurons following ischemic stroke, thereby clearing pathological inflammatory molecules released by dying cells and improving neurological outcome. This effect has been demonstrated up to 72 h following ischemic injury in mice [39], and notably, TREM2 expression has been shown to increase in the parenchyma following experimental SAH in a mouse model [54]. S1PR-independent signalling, such as via TREM2, may, therefore, act as another route for S1P-mediated neuroprotection.

Overall, these studies implicate a beneficial effect of parenchymal S1P and/or S1PR_1_ and S1PR_3_ signalling, either linked or independently, in rodent SAH models. It is important to bear in mind that all the evidence suggesting a beneficial role for S1P in the parenchyma is indirect and relies heavily on the specificity of the antagonists used. Parenchymal S1P levels were not measured, specifically to address whether S1P levels were decreased in the SAH model used (as was seen by Testai et al. [52]) and whether neuroprotection was accompanied by normalisation or further upregulation of S1P levels.

#### A Deleterious Role for S1P/S1PR Signalling in the Parenchyma

The putative neuroprotective effect of S1P is not supported by other studies which employ S1PR modulators. In rodent models, fingolimod has been shown to decrease inflammatory cytokines, preserve pial arteriolar response to vasodilators, reduce cerebral oedema and improve neurological outcome at up to 2 weeks following experimental SAH [31, 32]. RP001 hydrochloride, a structural analogue of fingolimod, decreased blood-brain barrier permeability, neuronal apoptosis and microglial/astrocytic activation, and improved neurological outcomes in a mouse model of SAH at 72 h [34]. In a mouse SAH model, S1PR_1_-specific inhibition with ponesimod prevented astrocytic transformation to the pro-inflammatory A1 subtype, decreasing the inflammatory response and neuronal apoptosis, and improving neurological outcome at 48–72 h [30]. Further evidence comes from non-SAH models. S1PR_3_-specific inhibition (CAY10444) in a mouse model of middle cerebral artery occlusion prevented microglial activation and M1 polarisation and was associated with decreased neurological injury at 24–72 h [37]. S1PR_3_ knock-out mouse astrocytes displayed a decreased inflammatory response to scratch injury in vitro compared to wild-type astrocytes [55]. Finally, in a mouse model of cerebral ischemia, direct injection of S1P into the parenchyma induced a neuroinflammatory astrocytic response 24 h post injection which was prevented by non-specific S1PR antagonism with fingolimod [56]. While these studies provide evidence that S1P signalling may be deleterious in the parenchyma, one needs to keep in mind that the effect of S1PR modulators may be independent of S1P/S1PR due to off-target effects.

### S1P/S1PR Signalling in the Parenchyma: Rationalisation of Evidence

**Fig. 4:**
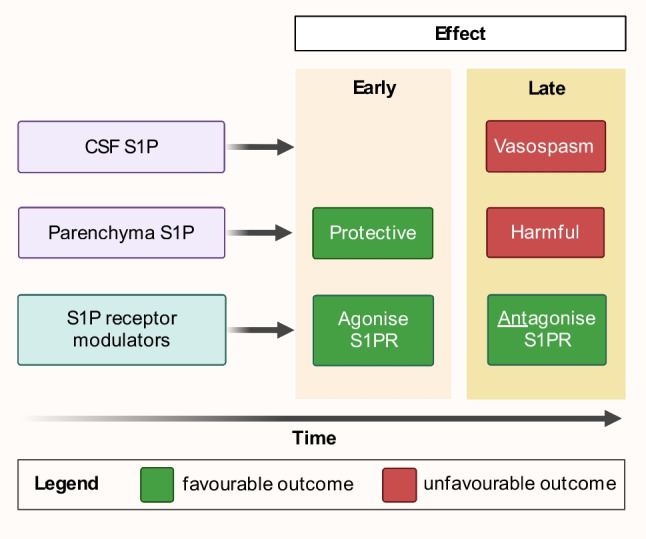
Proposed rationalisation of evidence regarding the role of sphingosine-1-phosphate (S1P) after aneurysmal subarachnoid haemorrhage. Parenchymal S1P is protective in the initial stages, and this effect is potentiated by the early agonist activity of S1P receptor modulators. In later stages, S1P is deleterious in both cerebrospinal fluid and parenchymal compartments. S1P causes vasoconstriction and, together with other mechanisms, may contribute to vasospasm of large arteries and also possibly of smaller arterioles in both cerebrospinal fluid and parenchymal compartments, respectively, which is delayed in onset. These delayed deleterious effects of S1P can be reversed by S1P receptor modulators, via development of sustained S1P receptor antagonism. Created with BioRender.com

In summary, while there is an agreement that parenchymal S1P plays a role after aSAH, there appears to be some contention as to the nature of this role. The most parsimonious explanation may relate to timing (Fig. 4). The majority of studies demonstrating a benefit of S1P signalling assessed outcome at 24 h or failed to show benefit at 72 h. However, this contrasts with studies showing that S1PR inhibition improved neurological outcome at up to 2 weeks. It may be that S1P signalling is initially neuroprotective and subsequently becomes harmful. It is important to note that the studies suggesting a detrimental effect of S1P utilised S1PR modulators, which were therapeutic in animal SAH models, and it is known that many of these drugs initially agonise receptors, followed by downregulation after internalisation.

There is a gap in our understanding of parenchymal S1P dynamics after aSAH, specifically how its concentration changes with time in the intracellular and/or extracellular space. The single study which demonstrated a reduction in S1P levels at 48 h [52] used rat brain homogenate, which does not differentiate between the intracellular and extracellular compartments. In addition, we do not know what happens to parenchymal S1P levels over time. Parenchymal S1P levels may increase beyond 48 h and exert a harmful effect in keeping with the previous observation that S1P effects may be different at low and high concentrations [57]. Hence, normalisation of parenchymal S1P signalling in the first few hours by S1PR modulators could be protective. However, if S1P levels continue to rise, parenchymal S1P signalling may shift to becoming harmful, such as by contributing to delayed microvascular spasm. At this time point, S1PR modulators exert a sustained S1PR antagonism, thereby providing a continual neuroprotective effect (Fig. 4).

## S1PR Modulators in Stroke and SAH Models

A number of S1PR modulators are currently licensed in the treatment of multiple sclerosis [58]. Fingolimod (FTY720) was the first licensed S1PR modulator. It is a prodrug which requires intracellular phosphorylation by SphK2 [59] prior to transport into the extracellular compartment by SPNS2 [60], where it can bind four of the five receptor subtypes (S1PR_1_, S1PR_3_, S1PR_4_, S1PR_5_). Second-generation S1PR modulators include siponimod, ponesimod and ozanimod. These modulators do not require phosphorylation in vivo and are more selective for certain S1PR subtypes. Siponimod and ozanimod bind S1PR_1_ and S1PR_5_, while ponesimod only binds S1PR_1_ [18]. Fingolimod and siponimod have both been shown to cross the BBB, whereas penetration of ozanimod and ponesimod is unknown [18].

S1PR modulation has been shown to decrease apoptosis, reduce inflammation/oxidative injury and preserve BBB integrity following CNS injury [61], including in ischemic and haemorrhagic stroke [9]. In ischemic stroke, there is evidence that fingolimod reduces inflammation and BBB impairment, leading to a reduction in ischemic lesion volume and improved clinical outcome in humans (*n* = 22) [62]. In a rodent model of intracerebral haemorrhage, fingolimod reduced cerebral inflammation and promoted clearance of blood breakdown products, improving neurological outcomes [39, 63]. In a small series of human intracerebral haemorrhage (*n* = 23), fingolimod reduced peri-haematoma oedema and improved clinical outcomes [64]. There is a growing body of evidence that S1PR modulators may also improve outcome following aSAH. As discussed above, the S1PR modulators fingolimod, RP001 hydrochloride and ponesimod have all been shown to improve neurological outcome and/or biological readouts in rodent models of SAH [30–32, 34] (Table 1).

The mechanism of action of S1PR modulators is complex. Brief initial agonist activity as the receptors are bound is followed by long-term inhibition of function once S1PRs are internalized and kept downregulated in the steady state [18]. Both fingolimod and ponesimod have been shown to cause transient agonism at S1PR_1_, followed by sustained functional antagonism through internalisation and failed recycling of the receptor [65, 66]. Fingolimod also displays initial agonist behaviour at S1PR_3_ [67, 68] accompanied by irreversible S1PR_3_ internalisation [69], again suggesting it acts as a functional antagonist at S1PR_3_ [70]. Importantly, this has been demonstrated in endothelial cell lines [70].

A potential neuroprotective mechanism of action for fingolimod in aSAH may include (1) reduction in neuroinflammation, apoptosis and BBB disruption through functional antagonism of S1PR_1_/S1PR_3_; (2) inhibition of cerebral artery S1P-induced vasoconstriction by functional antagonism of S1PR_3_; and/or (3) promoting cell survival and myelination through agonism of the oligodendrocyte S1PR_5_ [71, 72]. These potential mechanisms are supported by a study of fingolimod in a rat SAH model where neurological benefits were seen despite central blockage of S1PR_1_ and S1PR_3_ with VPC23019 [33], since this suggests redundancy. It is also possible that brief initial agonism at S1PRs by fingolimod capitalizes on the beneficial effect of parenchymal S1P in the early hours before vasospasm sets in; this is then followed by sustained antagonism to inhibit delayed vasospasm and the late deleterious effects of parenchymal S1P (Fig. 4).

In addition to S1PR-dependent mechanisms, fingolimod may exert anti-inflammatory effects via other pathways. A TREM2-mediated mechanism has been discussed above. Another example was demonstrated by a study of intracerebroventricular injection of fingolimod in a rat SAH model. Fingolimod-induced activation of protein phosphatase 2A resulted in upregulation of the anti-inflammatory protein tristetraprolin (TTP), reduced neuroinflammation and improved neurological function in a TTP-dependent manner [73].

In multiple sclerosis, fingolimod’s mechanism of action includes prevention of lymphocyte egress from peripheral lymphoid organs, thereby reducing the number of lymphocytes available to infiltrate the CNS [61, 74]. Neuroinflammation occurs following aSAH and has been implicated in cerebral vasospasm and outcome after haemorrhage [75]. There is limited literature specifically on lymphocytes following aSAH. Following an initial decrease in the first 24 h, blood lymphocyte count was found to increase, reaching a peak at day 7 after aSAH [76]. An elevated blood lymphocyte count has been implicated in worse clinical outcome and cerebral vasospasm following aSAH [77]. A systemic lymphocytic immunosuppressive effect may therefore act as another route by which fingolimod may influence outcome after aSAH. This mechanism is supported by experimental evidence in a rat SAH model where fingolimod reduced pial intravascular leucocyte adhesion [31].

## Clinical Translation

S1PR modulators may therefore be promising therapeutic agents to improve outcome after aSAH. As fingolimod targets a wider range of S1PRs including S1PR_3_, which appears to play an integral role in aSAH, and does not require dose titration (allowing rapid initiation of treatment), it may be the preferred therapeutic agent to improve outcome after aSAH. The S1PR_1_-specific modulator ponesimod has a number of potential advantages, although it requires dose titration. Firstly, it does not rely on phosphorylation in vivo like fingolimod and is, therefore, not dependent on SphK expression which introduces variability. Secondly, ponesimod has a much shorter half-life compared to fingolimod (32 h vs. 144–216 h) [18] which is particularly advantageous for acutely unwell aSAH patients who may require rapid discontinuation in the context of infection or cardiac complications (see below). Comparative preclinical studies of S1PR modulators are required to select the best drug to take forward to clinical trial.

Of particular importance following aSAH are cardiac side effects of S1PR modulators, since aSAH can lead to myocardial ischemia, arrhythmias and cardiomyopathy. S1PR modulators can cause first-dose chronotropic cardiac effects including bradycardia and conduction defects which are managed in MS either with first dose administration under observation (fingolimod) or with gradual titration of the S1PR modulator on an outpatient basis (siponimod, ozanimod and ponesimod) [18, 38]. aSAH patients would be in the right environment to initiate such a treatment, as inpatients under close monitoring when the treatment is started. Although S1PR_3_ has been implicated in the cardiac effects of S1PR modulators in mouse models, this effect appears relatively species specific, with S1PR_1_ now thought to mediate the majority of the cardiac effects in humans [38, 47]. Cardiac manifestations are therefore a potential side effect of all licensed S1PR modulators and are best managed by titration which is designed to limit the dose during the time that is needed for S1PR_1_ agonism at atrial myocytes to switch to functional antagonism. The newer S1PR modulators in clinical use for multiple sclerosis (siponimod, ozanimod and ponesimod) have a titration schedule with a duration of 6 to 12 days, but the length of this titration period would not have been subjected to specific study, since there is no immediate urgency to reach the maintenance dose in a chronic condition. Design of clinical trials of S1PR modulators in a SAH should therefore establish the optimum titration schedule, such that titration is not unnecessarily long in an acute condition, while still providing some time for atrial myocytes to adjust.

Of critical importance to clinical translation is a clearer understanding of the receptors which mediate the neurological sequelae of S1P after aSAH in humans, as this will govern which S1PR modulators are beneficial. S1PR_3_ appears to have a critical role for cerebral artery vasoconstriction in rodent models of SAH supporting the use of the non-specific receptor modulator fingolimod as it is the only modulator to target S1PR_3_. However, as discussed above, there are differences in the function of S1PR signalling between mice and humans, such as cardiac chronotropic effects, which may mean that S1PR_3_ does not mediate cerebral artery vasoconstriction in humans. Consequently, other S1PR modulators which do not target S1PR_3_ could be considered. This is further supported by the evidence implicating other S1PRs in outcome after SAH and the beneficial effects seen in animal trials of ponesimod (a S1PR_1_-selective modulator).

## S1P After aSAH: Mechanistic Summary

CSF S1P levels are undetectable in healthy individuals despite a plasma concentration of 1 μM [78]. This suggests active S1P uptake in the CNS, most likely via CFTR. After aSAH, CSF S1P concentration rises as a result of plasma-derived S1P as well as release by red blood cells and platelets within the blood clot. Tumour necrosis factor alpha causes downregulation of CFTR, and this may also contribute to high CSF S1P levels. In addition, high CSF S1P levels may inhibit uptake of S1P by residual CFTR in a positive feedback loop to maintain high CSF S1P levels. This inhibition of S1P uptake may explain why parenchymal S1P levels decrease while CSF S1P levels increase after aSAH. CSF S1P can cause vasoconstriction of arteries in the subarachnoid space and, together with other vasospastic agents which build up in the CSF over the first few days, can culminate in the onset of delayed cerebral artery vasospasm. Arterioles penetrating the cortex are also susceptible, since basal penetrating arterioles are accompanied by a pial sleeve containing CSF which ultimately mixes with the interstitial fluid. Within the parenchyma, evidence suggests that S1P is initially neuroprotective for a brief period, followed by a deleterious effect. It is not clear to what extent this may be a reflection of changing S1P levels in the parenchyma. We know that parenchymal S1P initially decreases after aSAH, and normalisation back to physiological levels may help recovery. As time passes, S1P levels in the parenchyma may rise, possibly by mixing of interstitial fluid with CSF and/or local production, and while such a postulated rise needs to be confirmed, we know that supraphysiological levels of S1P in the parenchyma induce microglial and astrocytic responses [56], so that parenchymal S1P effects are deleterious in the long term. The delayed rise in parenchymal S1P may also contribute to microvascular spasm. S1PR modulators seem to be tailored to treat this biphasic pathophysiological process since their initial agonist activity (potentially normalising S1P levels) is followed by a delayed and sustained antagonism (to inhibit the pro-inflammatory and other deleterious effects of S1P).

## Future Directions

A better understanding of how S1P levels change over time following aSAH in the CSF and parenchyma is required to assess the role of S1P signalling. This is particularly important in the parenchyma, although this compartment is technically more challenging to study. Lipidomic approaches using brain tissue sampled at the time of external ventricular drain insertion in humans, serial analysis in microdialysate and CSF in humans and a study of dynamic changes in the parenchyma of animal models of SAH would be valuable. A clearer understanding of the role and effects of parenchymal S1P following aSAH is required. If S1PR modulators are to be repurposed to improve outcome after aSAH, a comparative study in preclinical models is needed to guide a choice of the most suitable therapeutic agent, followed by clinical phase I study to develop a titration schedule that is short enough to provide therapeutic effects early after aSAH but long enough to minimise cardiac manifestations. Finally, newer agents, such as SPNS2 inhibitors [79], need to be tested in animal models of SAH.

## Review Criteria

PubMed was searched for articles published in English prior to July 2022 using the terms “sphingosine” AND “subarachnoid h(a)emorrhage”. All articles were reviewed by BG and IG. The reference lists of published articles were manually searched for additional articles.
